# A Novel Technique for Anterior Vaginal Wall Prolapse Repair: Anterior Vaginal Wall Darn

**DOI:** 10.1155/2013/198542

**Published:** 2013-02-12

**Authors:** Osman Köse, Hasan S. Sağlam, Şükrü Kumsar, Salih Budak, Öztuğ Adsan

**Affiliations:** ^1^Department of Urology, Faculty of Medicine, Sakarya University and Training and Research Hospital, 54100 Sakarya, Turkey; ^2^Beyaz Kent Sitesi, Beşköprü M. Girne C., 54100 Sakarya, Turkey

## Abstract

*Aim*. The aim of this study is to introduce a new technique, *anterior vaginal wall darn* (AVWD), which has not been used before to repair the anterior vaginal wall prolapse, a common problem among women. *Materials and Methods*. Forty-five women suffering from anterior vaginal wall prolapse were operated on with a new technique. The anterior vaginal wall was detached by sharp and blunt dissection via an incision beginning from the 1 cm proximal aspect of the external meatus extending to the vaginal apex, and the space between the tissues that attach the lateral walls of the vagina to the arcus tendineus fascia pelvis (ATFP) was then darned. Preoperation and early postoperation evaluations of the patients were conducted and summarized. *Results*. Data were collected six months after operation. Cough stress test (CST), Pelvic Organ Prolapse Quantification (POP-Q) evaluation, Incontinence Impact Questionnaire (IIQ-7), and Urogenital Distress Inventory (UDI-6) scores indicated recovery. According to the early postoperation results, all patients were satisfied with the operation. No vaginal mucosal erosion or any other complications were detected. *Conclusion*. In this initial series, our short-term results suggested that patients with grade II-III anterior vaginal wall prolapsus might be treated successfully with the AVWD method.

## 1. Introduction

Urinary incontinence and related symptoms due to pelvic organ prolapse (POP) are common conditions among females. The social, psychological, economical cost can be high [1, 2]. Almost 10% of women will, during their lifetime, need surgery for POP, urinary incontinence, or both. Of these, 30% will undergo two or more surgical procedures, presenting a challenge to gynecologist and urologist [3]. It has traditionally been treated with anterior colporrhaphy, which entails central plication of the fibromuscular layer of the anterior vaginal wall [4]. Recurrent anterior vaginal wall prolapse following conventional repair has been reported in more than 30% of the cases [5]. 

In an effort to improve outcomes in transvaginal prolapse repair, a number of graft materials have been introduced to complement, reinforce, or replace native tissue in reconstructive surgical procedures. The use of synthetic graft material for the repair of anterior vaginal wall prolapse has been limited by potential dangers related to the mesh (i.e., graft erosion, dyspareunia, pelvic pain, and infection). The lack of comparative data and anticipated incidence of graft-related complications (i.e., graft erosion and infection) has caused debate among surgeons about the necessity of graft use [6]. 

 For this reason, anterior vaginal wall darn (AVWD), which is carried out without mesh use, has been devised. It is (unlike colporrhaphy) unlikely to cause tension in the tissue; it is easy to apply, and (in contrast to mesh use) it does not corrupt the anatomical structures.

## 2. Materials and Methods

Forty-five patients were enrolled inthe study between the date August 2011 and March 2012, following written consent and approval from the local ethics committee. Patients ranged in age from 38 to 63 years (the median was 49) and had POP symptoms for the last nine months and stage II-III prolapse of the anterior vaginal wall. The preoperation evaluation consisted of a complete history, gynecological examination, cough stress test (CST), voiding diary, daily pad use, Q-Tip test, Incontinence Impact Questionnaire (IIQ-7), and Urogenital Distress Inventory (UDI-6) scores. Patients' symptoms were also evaluated by standard questions asked by the examining physician. The prolapse severity was additionally assessed using the POP Quantification (POP-Q) system adopted by the International Continence Society. Daily pad weight was used to quantify the patients' subjective complaints.

The exclusion criteria were: a previous pelvic or vaginal operation, predominant urge incontinence, pelvic or systemic infection, inguinal or vulvar abscess, pregnancy, urinary tract obstruction or renal insufficiency, pelvic pain (unrelated to prolapse), vaginal bleeding of unknown etiology, blood coagulation disorders, pelvic malignancy or previous radiation of the pelvic area, vaginal erosion or severe vaginal atrophy, vaginal or urethral fistula, and known allergy to the suture material. Patients requiring concomitant vaginal vault suspension such as sacrospinous ligament fixation, sacrocolpopexy for vaginal prolapse, uterine procidentia, laparotomy, or laparoscopy for any reason were also excluded. 

Postoperation workups, including POP-Q measurement, UDI-6, and IIQ-7, were performed for each patient 6 months after the AVWD procedure. Q-Tip test was also performed to evaluate the urethral hypermobility. Pre and postoperation questionnaire scores and POP-Q measurements were analyzed by using Wilcoxon signed-rank test. SPSS 12.0 software (Chicago, IL, USA) was used for data analysis. Differences were considered statistically significant when the *P* value was less than 0.05.

**Figure 1:**
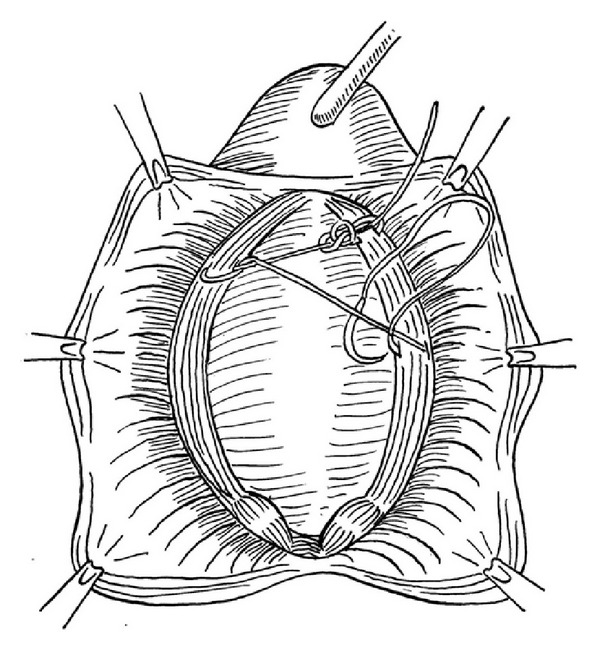
Slightly approximating continuous locking 2/0 polypropylene suture was placed inside the arcus tendineus fascia pelvis.

**Figure 2:**
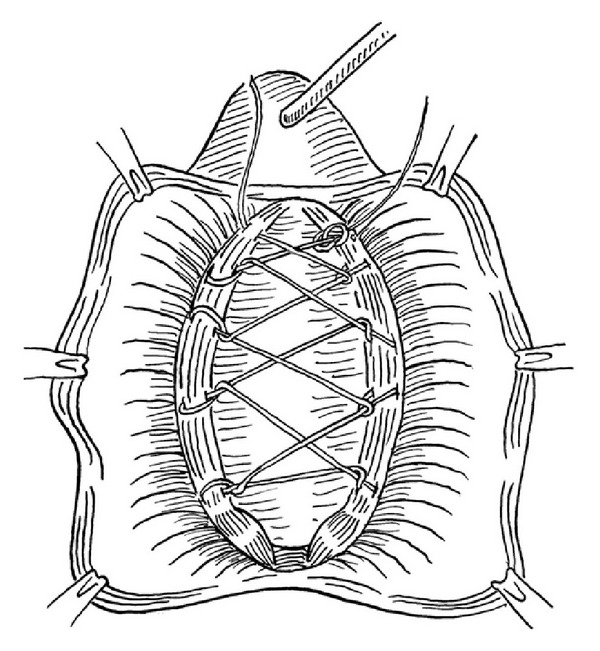
The suture was extended continuously to the distal aspect to form a darn.

**Figure 3:**
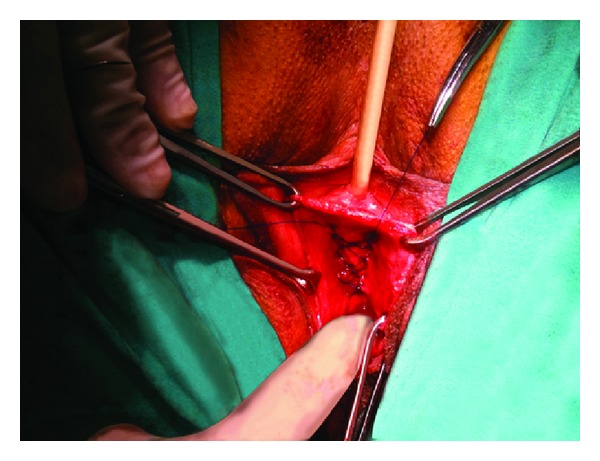
Surgical view of the ends of the suture.

Surgical technique: standard surgical technique was performed by the same surgeon. In the AVWD method, after insertion of an 18F urethral indwelling catheter, adequate normal saline was injected under the vaginal mucosa, thus providing a more comfortable dissection on an accurate plane with less hemorrhage. A midline incision was made beginning from the 1 cm proximal aspect of the external urethral meatus and extending to the vaginal apex. The anterior vaginal wall was detached from the urinary bladder beyond to the appearance of the anterior vaginal sulcus via a sharp and blunt dissection. Continuous locking 2/0 polypropylene suture was placed between the tissues that attach the lateral walls of the vagina to the arcus tendineus fascia pelvis (ATFP) beginning from the distal and extending to the proximal aspect as shown in Figure 1. The tissue used for anchoring, attaching the lateral walls of the vagina to the ATFP, is a condensation of connective tissue. The running suture was turned back from the cardinal ligaments without being tied and was extended continuously to the distal aspect to form a darn. The ends of the suture were tied together (Figures 2 and 3). The remnant vaginal mucosa was excised, and the mucosa was closed via a continuous absorbable suture, and a vaginal tampon was inserted.

## 3. Results

In total, 45 patients with anterior POP stage II-III were eligible to participate in the study. The baseline demographic and clinical parameters are shown in Table 1.

The median operating time was 40 minutes (the range was from 30 to 45 minutes); the average hospital stay was two days (the range was from 1 to 2 days), and the average time to void was 1.8 days (range 1-2 days). The preoperation and postoperation POP-Q measurements are shown in Table 2. Regarding the POP-Q, there were significant improvements at points Aa, Ba. Similarly, the UDI-6 and IIQ-7 scores dropped significantly after operation (*P* < 0.001) (Table 2). 

Moderate groin discomfort was the most prominent short-term postoperation problem, but this disappeared within 10 days of analgesic therapy. Six months after the surgery, all patients undertook a complete evaluation. All patients stated that urine leakage disappeared in all conditions. On examination, CST was negative, and vaginal examination appeared normal. Bladder ultrasound revealed no postvoiding residual urine. Symptom relief sixth months after operation is shown in Table 3.

## 4. Conclusions

The goal of treating POP is to improve quality of life rather than prolong survival. Therefore, when choosing a surgical method for anterior vaginal wall prolapse, it is important to consider possible complications as well as treatment outcome [7]. Although conservative treatment is a reasonable initial approach for urinary incontinence, surgical management is usually required for symptomatic grade II-III vaginal prolapse. While many operation methods have been developed to date, unfortunately, none could solve the problems resulting from POP. 

The issues of when, where, and how to perform the surgery, preferably as a single procedure, to provide the best outcome for the patient are in question. When selecting a surgical procedure for POP, pertinent factors include history of prior anti-incontinence surgery, sexual activity, coital incontinence, obesity, chronic increases in intra-abdominal pressure, mixed incontinence, or concurrent overactive bladder. 

In an effort to improve outcomes in transvaginal prolapse repair, a number of biologic and synthetic graft materials have been introduced since 1996 to reinforce or replace native tissue in reconstructive surgical procedures [8]. Results have been favorable, with anatomical success rates ranging from 59% to 94%. However, the use of mesh in vaginal repair procedures is still controversial [6, 9–11]. Uncontrolled studies have reported significant problems (e.g., dyspareunia, vaginal pain, mesh shrinkage, bladder erosion, fistula, mesh exposure, and infection) due to mesh use during vaginal prolapse surgery [12–16]. Vaginal mesh erosion is one of the most common complications of introducing synthetic material through the vaginal route. Different mesh types, different followup intervals, and even different definitions of success and failure have also contributed to the ambiguity in the erosion rates. No generally accepted “safety time zone” for mesh exposure or erosion has been recognized, and the complication can occur many years after the mesh placement. Young age and sexual activity are additional risk factors for mesh exposure [17]. 

Although there is increasing industry pressure for surgeons to adopt mesh-augmented repairs into their practice, and many surgeons are using the therapy liberally, health organizations such as FDA are warning urogynecologists and patients about using mesh materials in the treatment of POP [18, 19]. The committee for POP in the 3rd International Consultation on Incontinence concluded that there was insufficient data to make any definitive conclusion concerning the role of biologic or synthetic prosthetic materials in surgical procedures for primary or recurrent prolapse [20].

The data regarding the results of prolapse surgery remain nonhomogeneous. The success rate varies substantially depending on the technique used. Despite high anatomical recurrence rate, traditional anterior colporrhaphy, which entails central plication of the fibromuscular layer of the anterior vaginal wall, has been used for years for the treatment of POP [21, 22]. Although in this procedure pubocervical fascia is used to place plication sutures, histologic examination of the anterior vaginal wall has failed to demonstrate a separate layer of fascia between the vagina and the bladder [23]. On the other hand, as in hernia repair, tissue repairs carry high recurrence risk. The prime etiologic factor behind failures of tissue repair is the suturing together, under tension, of structures that are not normally in apposition. 

The rational for the darn procedure is to form a meshwork of nonabsorbable suture that is well tolerated by the tissues and fills the interstices with fibrous connective tissue providing buttress across the weakened area of the anterior vaginal wall. This technique is therefore a compensatory repair, and it becomes possible to repair anterior vaginal wall prolapse without distortion of the normal anatomy and with no suture line tension. A treatment, which is in accordance with the anatomical structure creating a hammock to reinforce the native support tissue, does not cause tension, and poses quite a low risk for vaginal mucosal erosion and urinary bladder injury, has been provided with this method. 

In this initial series, our short-term results suggest that grade II-III anterior POP may be treated successfully with AVWD technique with low complication rates. However, our AVWD technique does not seem as perfect as mesh technique according to the early postoperation appearance of anatomic site; it can be applied easily to young patients who consider about mesh erosion effect. 

However, more studies with long-term followups are ongoing to confirm these initial results.

## Figures and Tables

**Table 1:** Patient characteristics at baseline.

Characteristic	*n* = 45 (%)
Age	49 ± 11.4
Body mass index, kg/m^2^	
<30	28 (62.2)
30–40	11 (24.4)
>40	6 (13.3)
Parity	3 (0–6)
Topical estrogen	9 (20)
Hypertension	13 (28.8)
Smoking status	
Current	8 (17.7)
Former	11 (24.4)

**Table 2:** POP-Q, incontinence-related quality values.

	Before operation	After operation	*P* value
POP-Q measurements Aa (cm)	1.5 ± 1.2	−2.2 ± 0.9	<0.001
POP-Q measurements Ba (cm)	2.3 ± 1.6	−2.4 ± 1.1	<0.001
POP-Q measurements Ap (cm)	−2 ± 0.6	−2 ± 0.7	0.18
POP-Q measurements Bp (cm)	−2.57 ± 0.4	−2.6 ± 0.5	0.16
POP-Q measurements TVL (cm)	7.82 ± 0.32	7.95 − 0.53	0.54
POP-Q measurements C (cm)	−5.3 ± 1.5	−6.4 ± 1.3	0.041
UDI-6	8.9 ± 3.7	1.7 ± 1.1	<0.001
IIQ-7	11.8 ± 6.5	0.9 ± 0.6	<0.001
Q-TT	24.6 ± 5.1	8.3 ± 10.3	<0.001
Pad count (d)	4.2 ± 1.4	0.4 ± 0.8	<0.001
Residual urine volume (mL)	55.6 ± 10.6	47.4 ± 10.3	0.032

**Table 3:** Postoperative symptoms relief.

	Preoperative	Postoperative
Pelvic pressure	21	4
Sensation of a mass bulging into the vagina	33	—
Stress Urinary Incontinence	12	—
Coital incontinence	10	—
Difficulties in emptying the bladder	7	1
Mixed urinary incontinence	8	2
Dyspareunia	9	2
